# Deep Network-Assisted Quality Inspection of Laser Welding on Power Battery

**DOI:** 10.3390/s23218894

**Published:** 2023-11-01

**Authors:** Dong Wang, Yongjia Zheng, Wei Dai, Ding Tang, Yinghong Peng

**Affiliations:** State Key Laboratory of Mechanical Systems and Vibration, Shanghai Jiao Tong University, Shanghai 200240, China; wdshop@sjtu.edu.cn (D.W.);

**Keywords:** power battery, laser welding, two-branch network, coordinate attention, Hough transform, quality inspection

## Abstract

Reliable quality control of laser welding on power batteries is an important issue due to random interference in the production process. In this paper, a quality inspection framework based on a two-branch network and conventional image processing is proposed to predict welding quality while outputting corresponding parameter information. The two-branch network consists of a segmentation network and a classification network, which alleviates the problem of large training sample size requirements for deep learning by sharing feature representations among two related tasks. Moreover, coordinate attention is introduced into feature learning modules of the network to effectively capture the subtle features of defective welds. Finally, a post-processing method based on the Hough transform is used to extract the information of the segmented weld region. Extensive experiments demonstrate that the proposed model can achieve a significant classification performance on the dataset collected on an actual production line. This study provides a valuable reference for an intelligent quality inspection system in the power battery manufacturing industry.

## 1. Introduction

A power battery is one of the key components of new energy vehicles, and its quality determines the reliability and safety of the vehicle to a large extent. Laser welding is widely used in power battery manufacturing due to its advantages of high energy density, high precision, and precise control over the heat input [1,2]. However, on large-scale automatic production lines, on-site uncertainties such as material inhomogeneity, residual impurities, and parameter fluctuations increase the welding instability and easily lead to welding defects, which will seriously affect the quality and performance of power batteries [3,4]. At present, most of the post-welding quality evaluation of power batteries is mainly carried out by manual visual inspection, which is bound to cause low detection efficiency and high labor costs, making it difficult to meet the requirements of modern welding production for high efficiency and high quality. Therefore, an effective method is urgently needed to achieve automatic inspection of laser welding quality on power batteries.

In the past decades, traditional image processing methods have been broadly used in welding quality inspection [5,6,7]. Traditional visual inspection tends to manually extract geometric and textural features and build a learning-based classifier to predict the welding quality according to these features. For example, contour-based and OTSU threshold segmentation methods were used to extract keyhole features and weld width, and a back propagation neural network (BPNN) was trained to evaluate welding defects [8]. Cai et al. [9] extracted seven morphology features of keyholes and molten pools and compared the accuracy of several machine learning models (e.g., BPNN, radial basis function neural network, support vector regression) on welding quality prediction. However, such methods usually require strong expert knowledge and complex threshold settings, which are susceptible to environmental interference and have poor generalization performance and robustness.

Due to the powerful representation learning capability of convolutional neural networks (CNNs), deep learning-based visual inspection has shown excellent performance advantages in both accuracy and speed [10,11,12]. Visual quality inspection is generally divided into three stages: defect classification, defect localization, and defect segmentation. Defect classification models integrate feature learning and discriminative classifiers in one network to directly predict the welding quality category of the input image. Most of these models are established based on the improvement of existing classical networks, such as VGG [13], ResNet [14], MobileNet [15], etc. For instance, the pre-trained optimized VGG-16 model was established to accurately predict the welding defects on the safety vent of power batteries [16]. Furthermore, a lightweight model, SqueezeNet, was proposed to realize efficient and accurate detection [17], which is more suitable for scenarios with limited computational power. Transfer learning-based ResNet model was used to predict six spot welding defects in the automobile production site [18]. Defect localization models can obtain both accurate location and category information of welding defects, which can generally be divided into two-stage models represented by Faster R-CNN [19] and one-stage models represented by YOLO [20,21]. One-stage models pursue speed, while two-stage models emphasize accuracy. A Faster R-CNN model combining feature pyramid network was proposed, which can effectively detect small welding defects with weak contrast under complex backgrounds [22]. A bidirectional cross-scale fusion pyramid structure was introduced in YOLOv3 to facilitate the flow of information and enhance the fusion of multi-scale features for location and quality detection of small spot welding [23]. Compared to classification and localization, defect segmentation models enable a fine pixel-level localization of the defect region to obtain a more detailed description of the defect. Zhu et al. [24] presented a lightweight semantic segmentation method that can accurately segment laser welding defects of different shapes in real-time. A compressed U-shape network [25] was used for the quality inspection of laser welding in the battery production process, which has significant advantages in both speed and detection accuracy. By learning and fusing multi-scale semantic features of defects, the FPN-ResNet-34 network was proposed to predict the complete information, including defect category, boundary, and location, to achieve weld quality evaluation [26].

The success of deep learning stems from the construction of large annotated datasets. However, the amount of training data is difficult to meet for power battery production lines with strict qualification rate control. Therefore, to alleviate the problem of poor generalization performance due to insufficient training data, a two-branch network architecture is proposed for quality inspection. By learning two related tasks, weld segmentation and classification, the model can make full use of the useful supervisory information in images, thus reducing the data requirements for training the model. In addition, many defects and normal welds have high similarity in appearance, and it is difficult to find discriminative features in the images by using ordinary CNNs directly. The feature learning modules in the network should pay more attention to spatially distinguishable subtle features. Inspired by the visual attention mechanism, channel attention module [27], convolutional block attention module (CBAM) [28], and coordinate attention module (CAM) [29] have been proposed successively, which can learn critical information by assigning different weights to different regions in the images. In this paper, CAMs are introduced into the two-branch network architecture, which enables the model to effectively focus on the subtle features in the defective images that can represent its category. The accuracy and effectiveness of the proposed model are verified by the image dataset collected from the actual power battery production lines. Finally, edge detection and circular Hough transform are used to post-process the segmented weld image to obtain the weld information. In summary, the main contributions of the paper are outlined as follows:

(1) A two-branch network architecture is proposed, and weld region segmentation as a secondary task can effectively improve the classification accuracy of the model and reduce the demand for training data;

(2) The coordinate attention mechanism is introduced in the network to learn the more discriminative features between normal and defect;

(3) A framework combining deep network and conventional image processing is established to achieve efficient and accurate detection of laser welding quality.

The rest of the paper is organized as follows: The welding images dataset collected on the power battery production line is presented in Section 2. The proposed quality inspection framework is described elaborately in Section 3. In Section 4, the experimental results and discussions of the proposed model are comprehensively validated. Finally, the conclusion will be given in Section 5.

## 2. Overview of Welding Image Dataset

Power batteries are generally connected in series or parallel to form packs to obtain high capacity. When batteries are packaged, the connection between the battery poles and the adapter block needs to be implemented by laser welding. However, due to the high reflectivity of aluminum to the laser and the high tendency of parts to retain impurities such as stamping oil and cleaning agents, it is easy to lead to defects during the laser welding process. To carry out the model evaluation experiments, raw images of laser welding on battery packs are collected from an actual production line. To obtain high-quality images, an optical inspection system is embedded in the laser welder on the production line, consisting of an industrial camera and an LED-stabilized light source. Batteries are clamped on the assembly line by a bracket, and the light source is placed vertically above the assembly line. As the battery is welded and comes to rest in the camera’s field of view, its image is captured.

**Figure 1:**
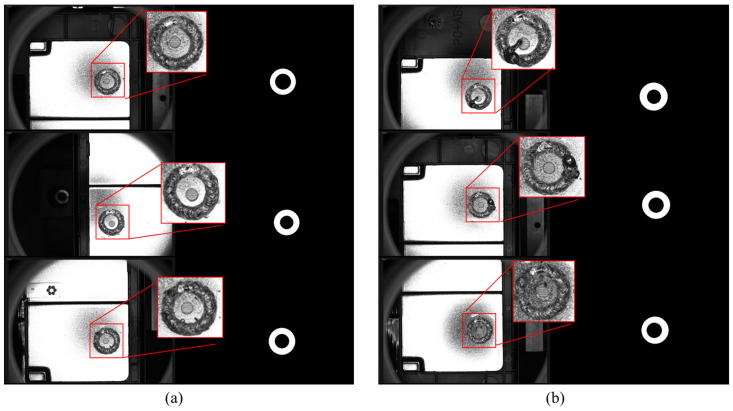
Some images with annotation masks in the dataset. (**a**) Qualified welds; (**b**)defective welds.

To acquire enough defective data for training, the expensive time cost is spent to construct the dataset. The dataset contains a total of 1268 images, of which 1040 are qualified welds, and 208 are defective welds. The resolution of raw images is 823 × 1129. To match the downsampling requirements of the network, zero padding is used for the border of images, and the image size is adjusted to 832 × 1152. In addition, the image resolution should be moderate; too large will affect the model inference speed, while too small will easily lead to reduced or even lost weld defect information. Finally, the image resolution is resized to 416 × 576 to obtain a trade-off between accuracy and speed. The proposed model follows a supervised paradigm that requires labeled data for training. In this paper, the open-source tool LabelMe is used to provide pixel-level annotations of the weld region and the presence or absence of weld defects for each image. Some examples of welding images with annotation masks are illustrated in Figure 1. It can be noticed that the contrast between welds and the background is not obvious, and the defects and qualified welds are relatively similar in appearance, which poses some challenges for quality prediction.

## 3. Proposed Framework

**Figure 2:**
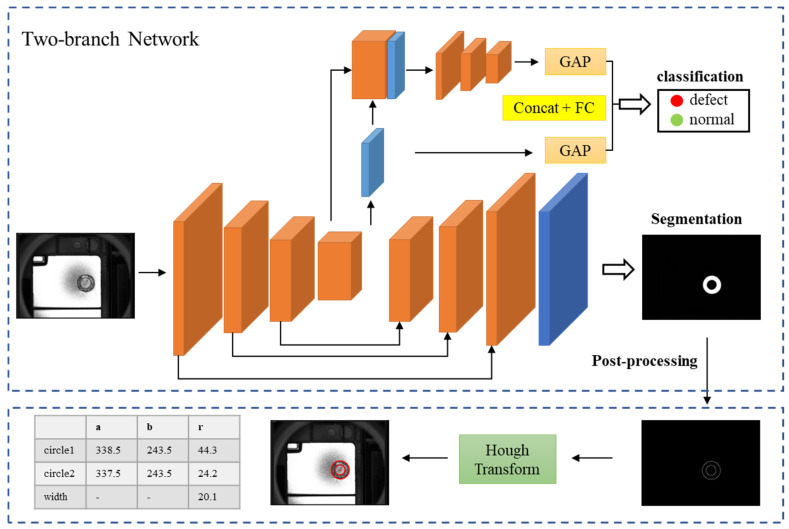
The proposed framework combining the two-branch network and conventional image processing. Note: (a,b) denote circle center coordinates and r denotes circle radius.

In actual production, besides the weld appearance quality, the weld width is also an important indicator. To this end, a quality inspection framework combining deep learning and conventional image processing is developed to identify weld defects while outputting weld information such as inner radius, outer radius, and width, as shown in Figure 2. In the framework, an end-to-end two-branch network is designed to alleviate the problem of large training sample size requirements for deep learning. The two-branch network is a multi-task learning method [30,31,32], which enhances model generalization performance by sharing feature expressions among related tasks. The proposed network includes a segmentation network for pixel-level localization of welded regions and a classification network for quality prediction. The segmentation network contains an encoder and a decoder, where the encoder extracts multi-scale features from the original image, and the decoder aggregates the feature maps and predicts the weld segmentation results. The classification network uses the feature maps obtained by the weld region-aware encoder to achieve accurate quality prediction. In this way, the two-branch network can make full use of the supervised information in each sample, increasing the effective number of training samples and reducing the risk of overfitting. Moreover, each network branch uses CAMs for feature learning to extract more discriminative features for subtle differences in weld defects. Finally, the segmented weld region is post-processed to extract the weld information by using edge detection and circular Hough transform [33,34].

### 3.1. Coordinate Attention Module

**Figure 3:**
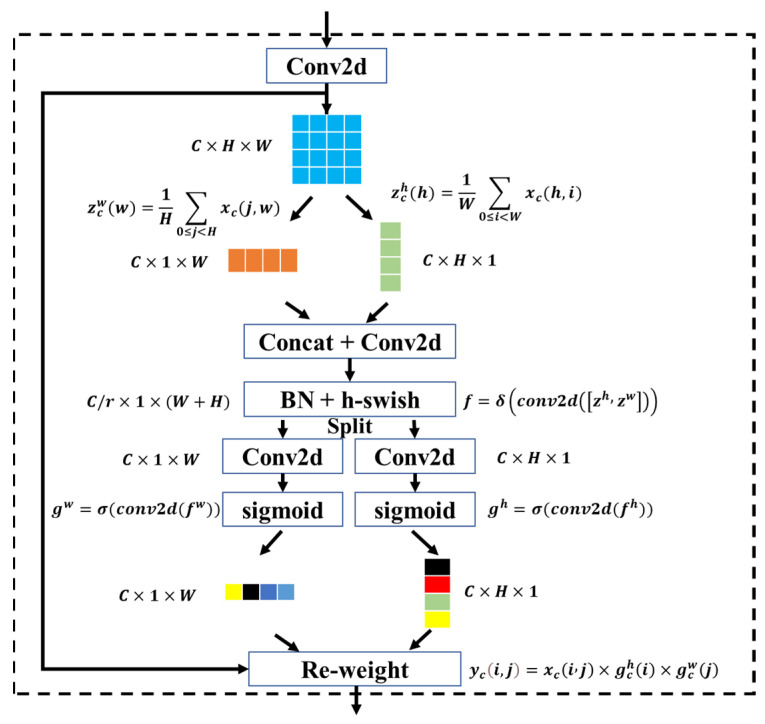
Diagram of the coordinate attention module (CAM). Note: the different colors in the bar below reflect the different weight values.

The proposed network uses CAMs instead of convolution modules in traditional CNNs, which allows better learning of weld detailed features and thus improves the detection accuracy of the model in welding defects. Unlike channel attention [27] and CBAM [28], CAM uses two average pooling operations to aggregate the input feature maps into two direction-aware features along the horizontal and vertical directions (Figure 3), thus alleviating the loss of location information caused by the direct use of two-dimensional global pooling. In this way, CAM can capture long-term dependencies along one spatial direction while maintaining accurate location information along the other direction. The two attention maps are then multiplied with the input feature map to enhance the representation of the object of interest; this allows for more accurate localization of the weld defect, which helps the model to better identify quality categories. The diagram of the CAM is illustrated in Figure 3.

Specifically, given the feature maps after two-dimensional convolution, represented as $X=[x_{1},x_{2},\ldots ,x_{c}]$. The average pooling of kernel size (H, 1) and (1, W) are then used to encode each channel along the horizontal and vertical directions, respectively. Thus, for the cth channel, the output at height h and width w can be expressed as:

(1) $$z_{c}^{h}\left(h\right)=\frac{1}{W}\sum_{0\leq i<W}x_{c}\left(h,i\right)$$

(2) $$z_{c}^{w}\left(w\right)=\frac{1}{H}\sum_{0\leq j<H}x_{c}\left(j,w\right)$$

The generated feature maps $z_{c}^{h}$ and $z_{c}^{w}$ are concatenated, combining the encoded information from the horizontal and vertical directions. Then, the concatenated feature maps are fed into a 1 × 1 convolution transform function, which applies a set of filters to extract relevant information from the input. Additionally, the incorporation of a Batch Normalization (BN) [29] layer following the convolution operation can effectively stabilize the input distribution of each layer within the network. This results in an improvement in training speed and a reduction in the overfitting issue.

(3) $$f=\delta \left(Conv2d\left(\left[z^{h},z^{w}\right]\right)\right)$$

where *δ* is the nonlinear activation function, and the h-swish function is chosen here. $f\in R^{C/r\times (H+W)}$ is the intermediate feature map, and *r* is the reduction ratio. Then, *f* is split into two independent tensors along the spatial dimension, $f^{h}\in R^{C/r\times H}$ and $f^{w}\in R^{C/r\times W}$. By using two 1 × 1 two-dimensional convolution transformations, $f^{h}$ and $f^{w}$ are converted into tensors with the same channel of the input X, respectively. The activation function σ used here is the sigmoid function.

(4) $$g^{h}=\sigma \left(Conv2d\left(f^{h}\right)\right)$$

(5) $$g^{w}=\sigma \left(Conv2d\left(f^{w}\right)\right)$$

The outputs $g^{h}$ and $g^{w}$ are extended and used as attention weights, respectively. Finally, the output of the CAM is denoted as $Y=[y_{1},y_{2},\ldots ,y_{c}]$, where each element $y_{c}\left(i,j\right)$ is reweighted by multiplying the original input feature $x_{c}\left(i,j\right)$ with the corresponding attention weights, resulting in the following:

(6) $$y_{c}\left(i,j\right)=x_{c}\left(i,j\right)\times g_{c}^{h}\left(i\right)\times g_{c}^{w}\left(j\right)$$

### 3.2. Two-Branch Network Architecture

**Figure 4:**
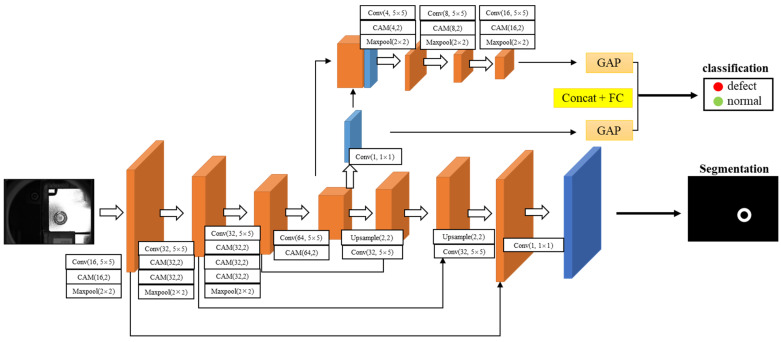
Detailed architecture of the two-branch network.

The detailed architecture of the two-branch network is presented in Figure 4, which consists of two parallel branch networks, a segmentation network, and a classification network. The segmentation network adopts a U-Net [35] similar structure, which consists of an encoder and a decoder, to effectively extract the multi-scale features and restore the image resolution. This branch segments the input image into weld and background regions and outputs the probability that each pixel belongs to the weld region. Specifically, the encoder contains convolutional layers, CAMs, and max-pooling layers. Each convolutional layer is followed by a batch normalization (BN) [36] layer and ReLU nonlinear function, where the combination of BN and ReLU enables more robust learning. After each 2 × 2 maxpooling operation, the image resolution becomes half of the original one. More importantly, several CAMs are used after the convolutional layer to enhance the model’s ability to perceive weld details. The main parameters in CAMs are the number of convolutional kernels and the reduction ratio *r*. Detailed parameters and the number of modules are shown in Figure 4. The encoder extracts feature maps with different resolutions and information levels from the image, while the decoder continuously increases the resolution of the feature maps by upsampling. The long connection from the encoder to the decoder allows the multi-scale feature maps in the encoder to be merged into the decoder, resulting in the more accurate performance of weld region segmentation. Finally, the original resolution and channel are restored by 1 × 1 convolution, where the sigmoid activation determines the probability that each pixel belongs to the weld region.

The classification branch and segmentation branch share the weight parameters of the encoder. Feature maps obtained from the encoder contain abundant weld region information, which effectively avoids the subsequent quality prediction being influenced by background. The weld quality features are further enriched by concatenating the output of the encoder with the single-channel mask feature maps. These feature maps are then fed into a series of combinatorial modules, each of which contains a convolutional layer with a 5 × 5 kernel size, a CAM, and a maximum pooling layer. The convolutional layers are followed by BN and ReLU, similarly, and the number of the convolutional kernel in each combinatorial module is 4, 8, and 16 in turn. As can be seen from Figure 4, the image resolution is further reduced, and the image is abstracted into a higher-level feature representation in the layer-by-layer operation of the classification network. The global average pooling (GAP) [37] layer reduces the redundancy and dimensionality of the features by calculating the average of all pixels within each channel of feature maps, thus reducing the overfitting possibility of the deep neural network. Finally, the GAP acts on both the high-level feature maps and single-channel mask, and their outputs are concatenated to 17 neurons, which are combined with a fully connected (FC) layer to obtain the final classification results.

The loss function of the two-branch network is a pivotal aspect of training the model effectively. In the proposed approach, the loss function consists of two distinct components, each serving a specific purpose, as presented in Equation (7). The first component employs the mean squared error (MSE) loss, which is utilized to measure the discrepancy between the predicted output of the weld region segmentation and the ground truth segmentation, as presented in Equation (8). The MSE loss ensures that the network accurately delineates the boundaries of the weld regions within the input data, minimizing the difference between the predicted and actual segmentations. The second component of the loss function incorporates the cross-entropy (CE) loss, as shown in Equation (9). Unlike the MSE loss, which assesses the similarity between continuous values, the CE loss is specifically designed for classification tasks. In our case, it addresses the quality classification aspect of the network’s output. By employing the CE loss, the network is trained to accurately classify the quality of the weld regions, assigning them to different classes based on predefined criteria (e.g., good quality, bad quality). The CE loss optimizes the network’s ability to correctly classify the quality of weld regions by penalizing incorrect predictions and encouraging accurate classification across different quality levels. By combining the MSE and CE losses within the overall loss function, the network’s performance can be simultaneously optimized in both weld region segmentation and quality classification. This multi-component loss function helps the network learn and adapt to the complex and interrelated tasks of accurately segmenting weld regions and classifying their quality, ultimately leading to improved performance and overall system effectiveness.

(7) $$L_{total}=L_{mse}+\lambda L_{ce}$$

(8) $$L_{mse}=\frac{1}{N}\sum_{i=1}^{N}{\left(y_{i}-x_{i}\right)}^{2}$$

(9) $$L_{ce}=-\frac{1}{N}\sum_{i=1}^{N}\left(y_{i}logx_{i}+(1-y_{i})log(1-x_{i})\right)$$

where $x_{i}$ and $y_{i}$ are the ground truth and prediction results, respectively. λ is the trade-off between two losses.

### 3.3. Post-Processing

The post-processing method utilizes edge detection and Hough transforms for circle detection in the input-segmented images. Edge detection is applied to extract the weld region contour using techniques like Canny [38] and Sobel [39]. Hough transform [33,34] maps the contour pixels from the image space to the circle parameter space, employing vote accumulation to identify the peak response in the parameter space; this allows for the determination of the circle parameters, which can be further employed to calculate the weld width and complete the evaluation of weld quality.

The flowchart of the Hough transform for circle detection is summarized in the algorithm (Algorithm 1). The algorithm initializes an accumulator array, A[a,b,r], with zeros to store the center points (a,b) and radius (*r*) of circles. The Canny operator is then used to detect the edge points in the segmented image, which indicates regions with significant changes in pixel intensity. Next, a nested loop is executed: the outer loop iterates from *r* = 0 to the diagonal length of the image in order to detect circles with different radii. For each edge pixel (x,y) in the image, the algorithm enters an inner loop that iterates over angles *θ* from 0 to 360 degrees, covering all possible angles around the edge pixel. Inside the inner loop, the algorithm calculates the potential coordinates for the circle center, *a* and *b*, using the current edge pixel (x,y) and radius *r*. These calculations involve subtracting the product of the radius and the sine or cosine of the angle *θ* from the edge pixel coordinates. Subsequently, the algorithm increments the accumulator array A[a,b,r] by 1 for each potential circle center and radius combination. This accumulation process counts the number of times a specific circle parameter combination passes through edge pixels in the image. After completing all iterations, the algorithm identifies the largest and second-largest values in the accumulator array, A[a,b,r]. These values correspond to the inner and outer circles of the weld. The parameters associated with these largest values represent the center coordinates (a,b) and the radius (*r*) of the detected circles.
**Algorithm 1:** Circle detection using Hough transformInput: the segmented images, image coordinates (x,y).Output: circle center coordinates (a,b), circle radius (r).1.Initialize: A[a,b,r]=0.2.Detect all edge points in the segmented image using the Canny operator.3.for r=0 to the diagonal length of the image4.         for each edge pixel (x,y) in the image5.                    for θ = 0 to 3606.                               a=x−r×cosθ7.                               b=y−r×sinθ8.                               A[a,b,r]=A[a,b,r]+19.Find the largest and second largest values of A[a,b,r], whose parameters correspond to the inner and outer circles of the weld.

## 4. Experiments and Discussion

This section provides a thorough exploration of various study aspects, including implementation details, evaluation metrics, experiment results analysis, and post-processing results. The section begins by discussing implementation specifics, offering readers a comprehensive insight into employed methodologies and techniques. It then focuses on evaluation metrics utilized to measure model performance. Subsequently, analysis of experimental results, including ablation experiments and sensitivity analysis of training samples, will be presented to demonstrate the performance advantages of the model in detecting weld quality. Lastly, the section concludes by analyzing post-processing results. This section presents a concise analysis and discussion, offering valuable insights into research methodology and outcomes.

### 4.1. Implementation Details

Experiments are conducted on a computer with i7-9700k CPU and NVIDIA GeForce GTX 1070ti GPU, and models are implemented based on the open-source Tensorflow deep learning framework. The input images and annotated masks are normalized before training, and the pre-processed dataset is divided into training and testing sets according to 4:1, where the category ratio is kept consistent. Moreover, to improve the generalization performance of the model, data augmentation is performed on the training samples using simple geometric transformations, including random rotation and flip. There is no scientific conclusion on the hyperparameter setting of the deep network. In this paper, the best hyperparameters are selected by using five-fold cross-validation, in which the training set is equally divided into five subsets, and each subset is used as the validation set in turn, and the remaining samples are used for training. The model performance is the average of the results of five training sessions and is used as the basis for hyperparameter selection. In this paper, the hyperparameter selection includes the number of CAM, parameters of the convolution layer, and so on. The weight λ of the two components in the loss function is set to 2 because the correct identification of defects is more important compared to weld segmentation in industrial scenarios. Adam [40] is used as the optimizer for model training with an initial learning rate of 0.001 and $\beta_{1}$ and $\beta_{2}$ of 0.9. A cosine scheduler with a warm-up is adopted to adjust the learning rate [41]. In the warm-up phase, the learning rate is gradually increased to a preset initial value to avoid the instability of the model parameters due to the large initial learning rate, after which the learning rate is decayed by the cosine annealing function, and the final learning rate is stabilized at 1 × 10^−5^. The training epoch and batch size are 200 and 8, respectively. The model parameters with the best performance on the validation set during the training are saved.

### 4.2. Evaluation Metrics

The classification branch mainly classifies images into two categories: defective and non-defective. Based on the confusion matrix in Table 1, metrics that are of concern to the production line are selected to quantify the classification performance, such as accuracy (Acc), miss alarm rate (MAR), and false alarm rate (FAR). Acc measures the overall performance of the model for defect and normal weld identification. MAR refers to the percentage of true defective welds that are missed by the model, which tends to lead to the outflow of defective welds. FAR indicates the percentage of welds predicted as defective that are misjudged by the model, which often results in wasted labor costs. The ideal situation is that the established quality evaluation method has high Acc, low MAR, and low FAR.

(10) $$Acc=\frac{TP+TN}{TP+FP+FN+TN}$$

(11) $$MAR=\frac{FN}{FN+TP}$$

(12) $$FAR=\frac{FP}{FP+TP}$$

For the segmentation branch, mean intersection over union (*mIoU*) is used as an evaluation metric, which quantifies the percentage of overlapping regions between predicted and actual annotation masks.

(13) $$mIoU=\frac{\left|A⋂B\right|}{\left|A\right|+\left|B\right|-\left|A⋂B\right|}$$

where $\left|A\right|$ and $\left|B\right|$ denote the number of pixels in predicted and ground truth images while $\left|A⋂B\right|$ denotes the number of common pixels in both images.

### 4.3. Experiment Results

The experimental results analysis consisted of an ablation experiment and sensitivity analysis of training samples. Ablation experiments are conducted, systematically removing study components to observe their impact on overall results. Additionally, the section investigates the model’s sensitivity to varying training sample sizes and examines how such variations affect performance.

#### 4.3.1. Ablation Experiment

In this section, ablation experiments are conducted for each component of the proposed network, and the effectiveness of each component is analyzed by comparing its performance on the test set. The detailed results are presented in Table 2. The advantages of the two-branch structure are first investigated. From the comparison results in Table 2, it can be found that the performance indicators of the two-branch structure are significantly improved compared to the single-branch. For example, compared to the single-branch model with CAMs, the proposed model improves by 1.3%, 5.2%, and 2.9% in *Acc*, *MAR*, and *FAR*, respectively; this indicates that the model can learn a more accurate feature representation of weld through the joint training of two related tasks. In addition, the effect of coordinate attention on classification performance is also studied. After introducing CAMs into the feature learning module of the proposed network, the Acc increased by 0.4% compared to the two-branch CNN, while *MAR* and *FAR* decreased by 1.6% and 1.0%, respectively. Similarly, compared with the original CNN, the model with CAMs has improved in all metrics. These results indicate that CAMs have a stronger ability to capture subtle features of defects compared to ordinary CNN.

More importantly, in terms of the magnitude of the performance improvement, the two-branch structure is better than coordinated attention. For example, the performance improvement is 0.2%, 0.6%, and 1.0% after introducing CAMs into CNN, while the improvement can reach 1.1%, 4.2%, and 2.9% when applying the two-branch structure to CNN. By establishing two related tasks, segmentation and classification, the two-branch network can highlight the weld semantic information in the training process under the full use of supervised information in the images. This structure provides better results than the direct use of coordinated attention, and the combination of both enhances the classification performance of the weld quality.

#### 4.3.2. Sensitivity to the Number of Training Samples

**Figure 5:**
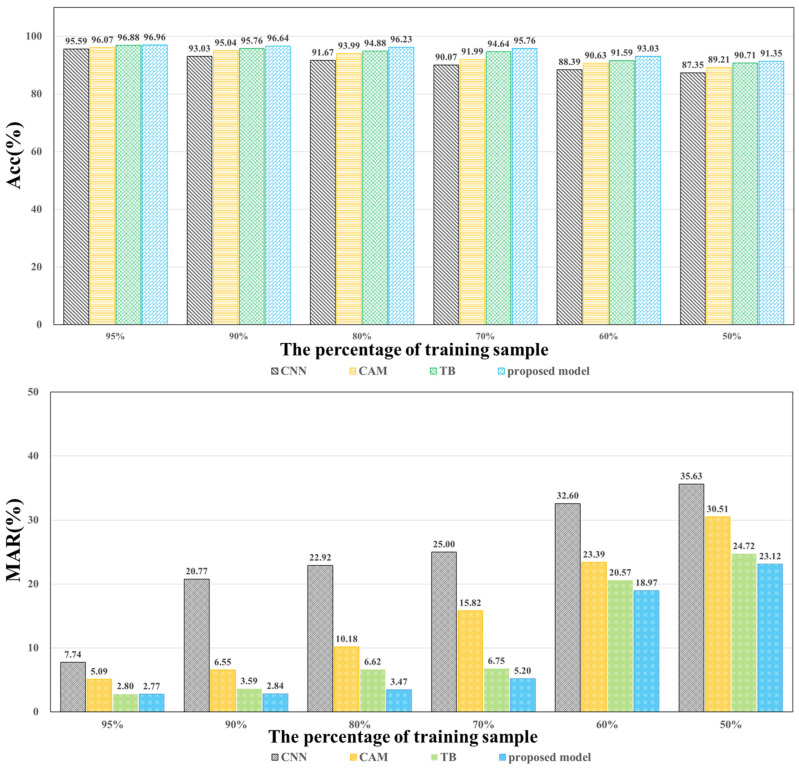

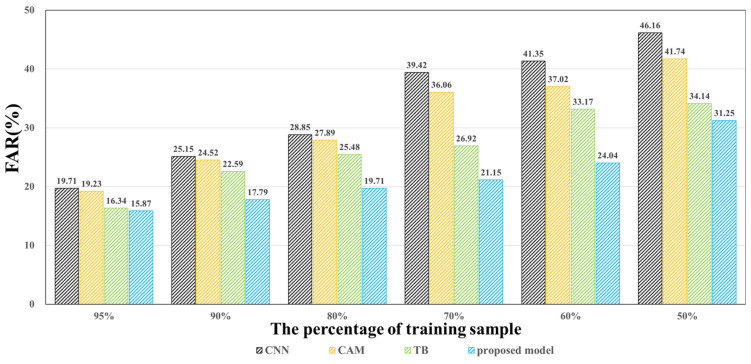
Performance metrics at a varying number of training samples.

As mentioned above, it is very expensive to collect a large amount of defect data on an actual production line. Therefore, the impact of a smaller training sample size on model performance is also investigated. The training set is randomly sampled for the experimental study by 95%, 90%, 80%, 70%, 60%, and 50%, respectively. The same category ratio is maintained as the original dataset in the sampling process. The rest of the settings follow the same training and testing procedure as the previous experiments. The detailed results are presented in Figure 5. It can be found that compared with the single-branch network, the two-branch structure model is not sensitive to the number of training samples. The performance of the proposed network model still remains 95.76% *Acc*, 5.20% *MAR*, and 21.15% *FAR* when the number of training samples is 70% of the original. When using a smaller dataset (50%), the Acc decreases by 6.55% compared to the original one, while *MAR* and *FAR* increase by 22.02% and 19.75% in the test set. On the contrary, the impact of decreasing the training sample size on the single-branch model is significant, with a rapid decrease in all performance metrics. The performance is poor when the number of training samples is small. For example, *Acc*, *MAR*, and *FAR* of CNN are 87.35%, 35.63%, and 46.16%, respectively. Compared to the original model, its *Acc* decreased by 9.05%, while *MAR* and *FAR* increased by 28.73% and 30.76%, respectively. Such a comparison demonstrates that the two-branch network has a significant effect on alleviating the training sample demand. Overall, the experimental results show that the method can still maintain a good, stable performance with a small number of training samples.

### 4.4. Post-Processing Results

**Figure 6:**
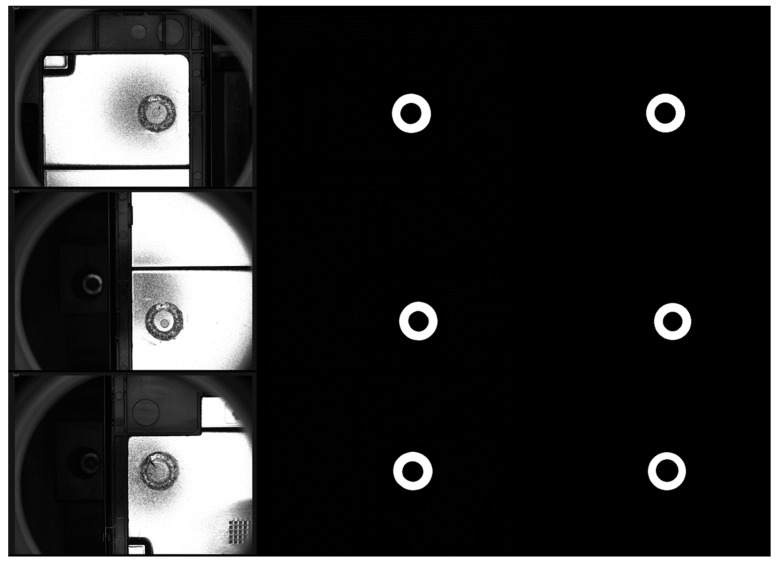
Some examples of segmentation results of the proposed model. From left to right are the raw welding images, the segmentation results of the proposed network, and the ground truth.

Figure 6 shows the weld segmentation results obtained by the proposed two-branch network and the corresponding annotation masks. It can be intuitively illustrated that the model can achieve the pixel-level localization of the weld relatively well. The *mIoU* results in Table 2 also quantitatively show that the model can segment the weld region accurately from the raw welding images.

**Figure 7:**
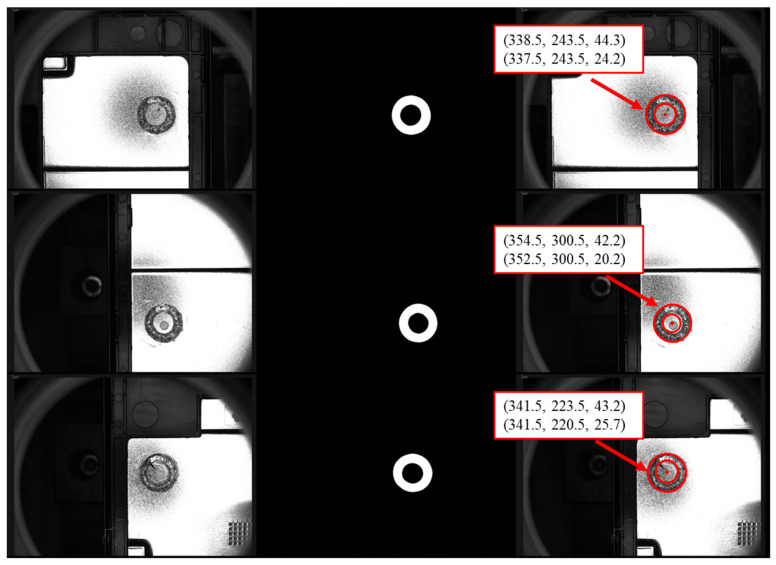
Some examples of the post-processing method. From left to right are the raw images, segmented images, and extracted parameters (a,b,r).

After obtaining the binary segmentation images, the post-processing method combines the Canny and circular Hough transform to achieve the extraction of the internal and external circular parameters. The weld width information can be easily calculated from the inner and outer circle parameters, which are measured in pixel lengths. Some results of post-processing methods are shown in Figure 7. It can be seen that the framework proposed in this paper can effectively extract the weld region parameters from the welding images on power batteries. In addition, the accuracy of the welding parameter extraction relies heavily on the results of the segmentation model in the previous section. When the segmentation model does not perform well, it will seriously deteriorate the effect of the post-processing algorithm.

## 5. Conclusions

The automatic detection of laser welding quality in power batteries is crucial for ensuring the safety performance of new energy vehicles. This paper proposes a framework that combines deep network and conventional image processing techniques to achieve efficient and accurate detection of laser welding quality.

(1) The framework consists of a two-branch network, comprising a segmentation network trained with pixel-level labels and a decision network built on top of it to predict the presence of weld defects. The joint learning of these two tasks effectively enhances the accuracy of the features and the generalization ability of the model;

(2) To effectively learn the distinguishing features between normal welds and defective welds, the detection network incorporates a coordinate attention module. Additionally, a post-processing method based on the Hough transform is employed to extract geometric information about the segmented weld region, including the position of the weld’s center and its width;

(3) An extensive evaluation of the model is conducted on a dataset collected from a real production line. The evaluation encompasses ablation experiments and investigates the impact of the training sample size. The experimental results demonstrate that both the coordinate attention module and the two-branch structure significantly improve the model’s performance for weld detection, as indicated by metrics such as Acc, FDR, and MAR.

This study contributes a new approach to quality prediction with promising implications for battery pack welding. Although the present work is focused on a specific quality inspection application, it can be believed that the underlying principles and methodologies can be adapted and applied to similar scenarios. For instance, in body spot weld quality inspection, where the defect rate is low on the production line, the accuracy of the model in recognizing defective welds can be improved by utilizing the defective area of spot welds as an auxiliary task. In future work, the feasibility and effectiveness of extending the proposed framework will be explored for other manufacturing quality inspection tasks that have similar dataset constraints.

## Figures and Tables

**Table 1:** Confusion Matrix.

Confusion Matrix		Prediction	
		Normal	Defect
True	Normal	True Negative (*TN*)	False Positive (*FP*)
	defect	False Negative (*FN*)	True Positive (*TP*)

**Table 2:** The comparison results from different combinations of CAM and two-branch (TB).

CAM	TB	*Acc*	*MAR*	*FAR*	*mIoU*
—	—	0.964	0.069	0.154	—
√	—	0.966	0.063	0.144	—
—	√	0.975	0.027	0.125	0.926
√	√	0.979	0.011	0.115	0.957

Note: “—” means that the component does not exist in the network, while “√” means that the component is introduced.

## Data Availability

Not applicable.
